# Periapical lesion healing in osteoporosis patients: an explorative retrospective, controlled pilot cohort study

**DOI:** 10.1007/s00784-025-06739-x

**Published:** 2026-03-09

**Authors:** Thomas Gerhard Wolf, Senta Sophia Geiger, Benjamin Briseño-Marroquin, Guglielmo Campus, David Donnermeyer, James Deschner, Anna Damanaki

**Affiliations:** 1 Department of Restorative, Preventive and Pediatric Dentistry, School of Dental Medicine, University of Bern, Bern, Switzerland; 2 Department of Periodontology and Operative Dentistry, University Medical Center of the Johannes Gutenberg University Mainz, Augustusplatz 2, 55131 Mainz, Germany; 3 Department of Cariology, Institute of Odontology, Sahlgrenska Academy, University of Gothenburg, Gothenburg, Sweden

**Keywords:** Bone regeneration, Endodontics, Healing, Osteoporosis, Periapical periodontitis, Retrospective

## Abstract

**Aims:**

Osteoporosis is a systemic disease characterized by reduced bone density. The aim of this exploratory, retrospective controlled pilot study following STROBE guidelines, was to descriptively assess periapical lesion healing following endodontic treatment in patients with osteoporosis compared with matched healthy controls and to generate hypotheses for future studies.

**Methods:**

Digital X-ray images of patients who underwent endodontic treatment at a German university dental clinic between January 2011 and December 2020 were evaluated. Patients with radiographically visible periapical lesions, a follow-up radiograph obtained 6–12 months after treatment, and a documented diagnosis of osteoporosis were included. Changes in periapical lesion size and Periapical Index (PAI) scores were assessed and compared with an age- and tooth-group-matched control cohort. Given the limited sample size, analyses were considered exploratory.

**Results:**

Out of 103,385 screened patients, 13 patients with osteoporosis (4 male, 9 female) fulfilled all inclusion criteria and were matched to 13 healthy controls. Both groups demonstrated a reduction in periapical lesion size over time (mean change: -2.47 mm in the osteoporosis group and − 1.90 mm in controls). A mean improvement of one PAI grade was observed in both cohorts. No statistically significant group differences were detected.

**Conclusions:**

Within the limitations of this exploratory pilot study, no statistically significant differences in radiographic periapical healing were observed between patients with osteoporosis and matched healthy controls. Due to the limited sample size and the resulting limited statistical significance, these findings should be interpreted with caution, and further studies are needed to clarify the possible influence of osteoporosis on endodontic healing.

**Clinical relevance:**

With demographic change, the potential influence of osteoporosis on endodontic healing is of increasing clinical interest. This study highlights both feasibility and methodological challenges when addressing this question in retrospective clinical cohorts.

## Introduction

With demographic change, osteoporosis will become increasingly important alongside other age-related chronic diseases due to the expected rise in the number of cases [1]. Osteoporosis is considered one of the most widespread bone diseases. It is estimated that approximately 200 million women worldwide are affected, of whom approximately 10% are aged 60, 20% are aged 70, 40% are aged 80, and up to two-thirds are aged 90 [2]. One in three women after menopause and one in five men over the age of 50 are affected by the disease [3]. In Germany, an estimated 6–8 million people suffer from osteoporosis [4, 5]. Osteoporosis is characterized by low bone density and microarchitectural deterioration of bone tissue. These factors occur particularly in older people [6]. However, people are not only living longer, but they are also keeping their teeth longer thanks to ever-improving preventive dental care [7]. As a result, endodontology is becoming increasingly important for this population group. While several studies have already investigated the interaction between periodontal disease and osteoporosis [7–9], the question of a connection between osteoporosis and the healing process after adequate root canal treatment remains largely unanswered. A recent systematic review indicates a potentially increased prevalence of periapical lesions in osteoporotic patients compared to non-osteoporotic patients, but the evidence is considered very limited due to methodological weaknesses and an overall low study quality [10]. In addition, data from a large retrospective cohort study show that osteoporotic patients have significantly more periapical lesions, but treatment with bisphosphonates may be associated with a reduced lesion prevalence [11]. The aim of this explorative retrospective, controlled pilot cohort study was therefore to explore the potential influence of osteoporosis on the healing of periapical periodontitis following endodontic therapy and to generate hypotheses as well as preliminary data to inform the design of future adequately powered studies.

## Methods

### Inclusion and exclusion criteria

This retrospective, controlled cohort study was approved by the Ethics Committee of the Rhineland-Palatinate Chamber of Physicians (2020–15180-retrospective, July 30, 2020). This study was conducted in accordance with the STROBE guidelines, which are designed to improve the reporting of observational studies in epidemiology. Inclusion criteria were osteoporotic disease and available X-ray images at the time of the necessary endodontic treatment (diagnostic image, baseline) and at least 6 months, up to a maximum of 12 months thereafter (control image, follow-up) in order to be able to assess the healing process of a periapical endodontic lesion. Patients were excluded if the follow-up X-rays were taken less than 6 months after the diagnosis, if the tooth was extracted, or if the X-ray image was insufficient for evaluation. The treatment documentation was reviewed independently by two observers (S.G., A.D.).

### Technology and software

The Visident system (BDV Dental GmbH & Co. KG, Holzwickede, Germany) of the University Medical Center of the Johannes Gutenberg University Mainz (Germany) was used to filter out those patients who received endodontic treatment during the relevant period. The relevant data were recorded in a database in Microsoft Office Excel 2016 (Microsoft Corp., Redmond, WA, USA) and processed using a PC. The corresponding X-ray images of the included patients were measured using the Sidexis XG program (neXt Generation, Version 2.63 2016, Sirona Dental System GmbH, Bensheim, Germany).

### Evaluation of the X-ray images

The assessment and measurement of radiographic images were conducted in a darkened room, free from distracting light sources. Prior to evaluation, monitor brightness and contrast were calibrated according to SMPTE image standards to ensure consistent image quality. Each radiograph was assessed for diagnostic suitability, and images of insufficient quality were excluded from the dataset. All radiographs were independently evaluated by two examiners (S.G., A.D.) in collaboration with an experienced radiographic assessor. Given the inherent subjectivity of radiographic interpretation, the examiners underwent prior calibration using a set of twelve reference radiographs provided by the experienced assessor (B.B.M.). In cases of uncertainty or disagreement, findings were jointly reviewed and consensus was reached through discussion. The measurements were performed using the measurement tool included in the Sidexis XG program (neXt Generation, Version 2.63 2016, Sirona Dental System GmbH, Bensheim, Germany). The length and width of the visible periapical lesion were measured in the baseline X-ray image and in the follow-up X-ray image of the included patients. The periapical index (PAI), according to Estrela et al. (2008) served as the basis for the evaluation [12]. To reduce potential sources of bias, standardized inclusion criteria were applied, radiographic assessments were performed independently by two calibrated examiners, and control patients were matched for age and tooth group.

It is classified as follows: 0: Normal periapical bone structure, 1: Diameter of periapical radiolucency of 0.5–1 mm, 2: Periapical radiolucency diameter of 1–2 mm, 3: Periapical radiolucency diameter of 2–4 mm, 4: Periapical radiolucency diameter of 4–8 mm, and 5: Periapical radiolucency diameter of > 8 mm.

### Control group

When selecting patients for the control group, which likewise comprised 13 individuals, the absence of systemic diseases was ensured. To achieve comparability between groups, control patients were matched to the test group based on age and type of affected tooth. Age matching was considered acceptable if the difference between matched individuals did not exceed ± 5 years at the time of treatment. As exact matching of individual teeth proved challenging, teeth were categorized into predefined tooth groups; a match was deemed sufficient when both individuals had a tooth affected within the same group. The teeth were divided into groups as follows: Anterior maxillary teeth (13–23), maxillary premolars (14–15, 24–25), maxillary molars (16–18, 26–28), anterior mandibular teeth (33–43), mandibular premolars (34–35, 44–45), and mandibular molars (36–38, 46–48).

### Statistical analysis

Following radiological evaluation, the collected data were statistically analyzed using the statistical software IBM SPSS Statistics 27.0 (SPSS Inc., Chicago, USA). Comparisons between the two groups regarding the ‘size of periapical lesion’ and the corresponding ‘change score’ were performed using Mann–Whitney U tests.

## Results

In this explorative retrospective, controlled pilot cohort study, patient records from January 2011 to December 2020 were screened, comprising a total of 103,385 individuals, of whom 2,998 underwent endodontic treatment. Among these, 841 were systemically healthy, 1,378 presented with various systemic conditions, and 211 were excluded due to incomplete treatment documentation. A subgroup of 568 patients was identified with diagnoses of osteoporosis, rheumatoid arthritis, thyroid dysfunction, or cardiovascular disease. Six additional patients were excluded: in four cases, follow-up radiographs were obtained in less than six months post-treatment, one tooth had been extracted, and one case was excluded due to insufficient radiographic quality for assessment. Ultimately, 13 patients diagnosed with osteoporosis (four male, nine female) met the inclusion criteria and were enrolled in the study, including radiographic follow-up. The age of these patients ranged from 50 to 90 years. The most frequently treated teeth were maxillary anterior teeth (38.46%) and mandibular molars (23.08%).

### Pre- and post-therapeutic periapical findings

**Fig. 1:**
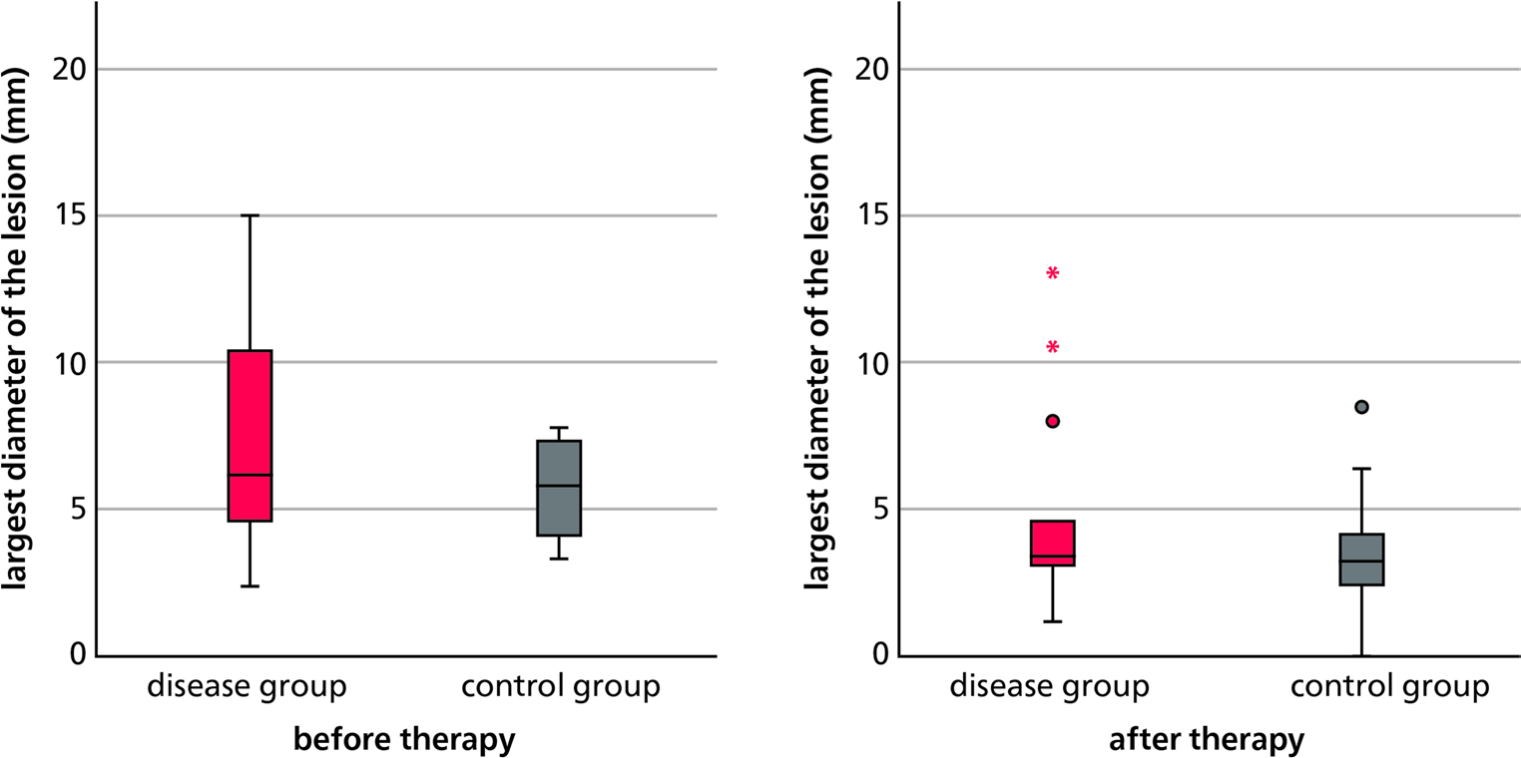
Sizes of periapical lesions (in mm) before and after endodontic treatment in the osteoporosis (disease) and control groups

**Fig. 2:**
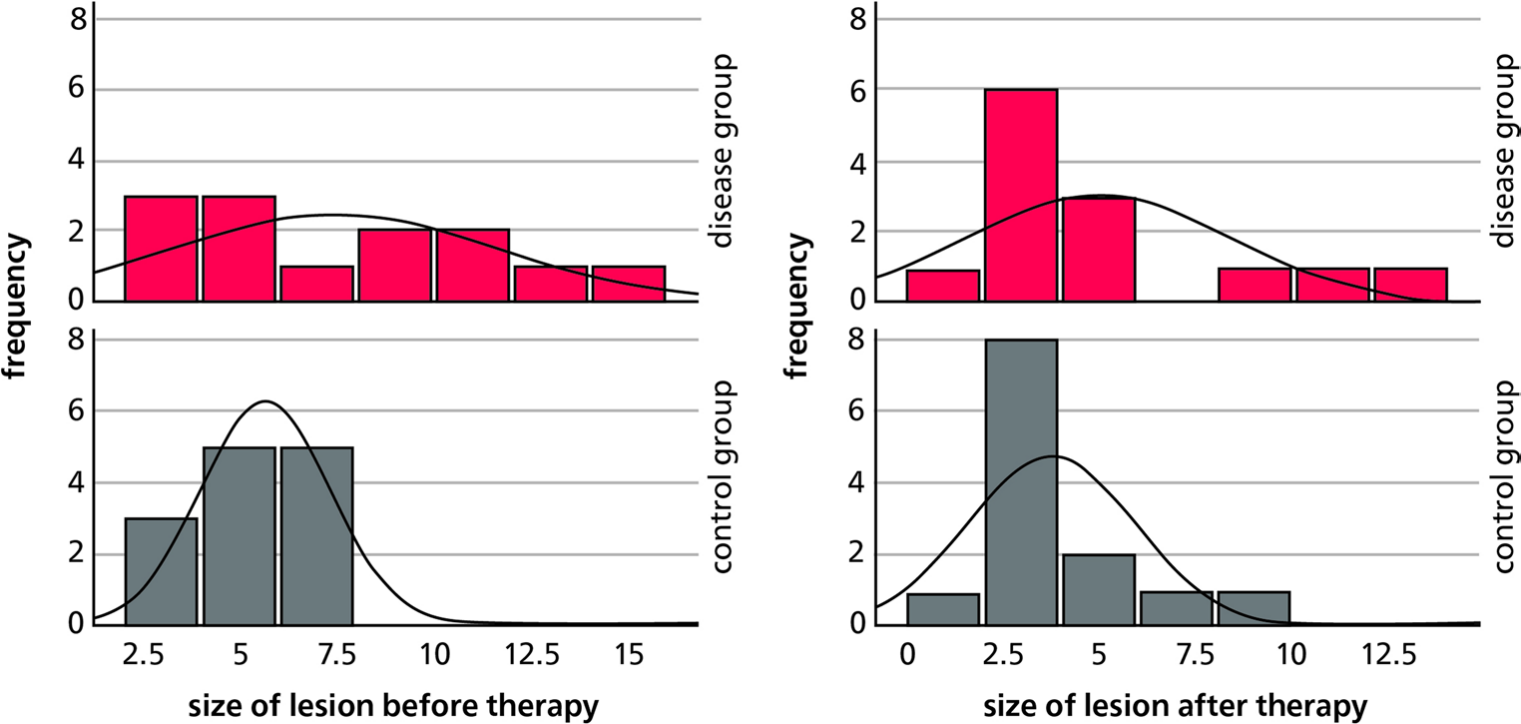
Sizes of periapical lesions (in mm) before and after endodontic treatment in the osteoporosis (disease) and control groups

To assess the extent of periapical pathology, the largest diameter of each lesion was measured. In the osteoporosis group, preoperative lesion sizes ranged from 2.36 mm to 15.03 mm, with a mean of 7.47 mm (SD ± 4.19). In contrast, the control group presented with smaller lesions prior to treatment, ranging from 3.30 mm to 7.77 mm, with a mean of 5.67 mm (SD ± 1.65). Although the medians were nearly identical in both groups, the variance was considerably greater in the osteoporosis group. A minimum follow-up of 6 months and a maximum of 12 months after endodontic treatment, the mean lesion diameter had decreased to 5.00 mm in the osteoporosis group and to 3.78 mm in the control group. The respective standard deviations were 3.45 and 2.17 (Fig. 1). Lesion size distributions in both groups are also illustrated as histograms in Fig. 2.

For categorical evaluation of the periapical status, the Periapical Index (PAI) according to Estrela et al. [12] was used. This index ranges from Grade 0 (intact periapical bone structure) to Grade 5 (extensive lesion > 8 mm). Prior to treatment, Grade 5 lesions were observed exclusively in the osteoporosis group (23.08%). Grade 4 affected 4 teeth (15.38%) in the osteoporosis group and 10 teeth (38.46%) in the control group. Grade 3 was observed in 3 teeth (11.54%) in each group.

Following treatment, Grade 5 lesions were recorded in 3 teeth (11.54%) in the osteoporosis group and in 1 tooth (3.85%) in the control group. Grade 4 affected 3 teeth (11.54%) in both groups. Grade 3 was the most common finding in both groups, involving 6 teeth (23.08%) in the osteoporosis group and 8 teeth (30.77%) in the control group. Grade 2 was observed in 1 tooth (3.85%) exclusively in the osteoporosis group. Grade 1 was not recorded in either group, and Grade 0 was observed in 1 tooth (3.85%) in the control group.

Statistical analysis using the Mann–Whitney U test revealed no significant difference in lesion size between the groups at baseline (*p* = 0.418). Similarly, no significant difference was found within the osteoporosis group after treatment (*p* = 0.479).

### Comparison between the osteoporosis and control groups

To evaluate treatment outcomes between the two cohorts, the difference in periapical lesion size before and after endodontic therapy—referred to as the change score—was calculated for each group. This metric reflects the extent of lesion size reduction or enlargement over time, expressed in millimeters. On average, periapical lesions decreased by 2.47 mm in the osteoporosis group and by 1.90 mm in the control group. When translated into PAI scores, both groups demonstrated an average improvement of one grade (Table 1).

**Table 1:** Change scores in both groups

	Disease group	Control group
Mean change score (absolute values) [mm]	-2.47	-1.9
Median change score (absolute values) [mm]	-1.33	-2
Mean change score PAI	-0.62	-0.62
Median change score PAI	-1	-1

**Fig. 3:**
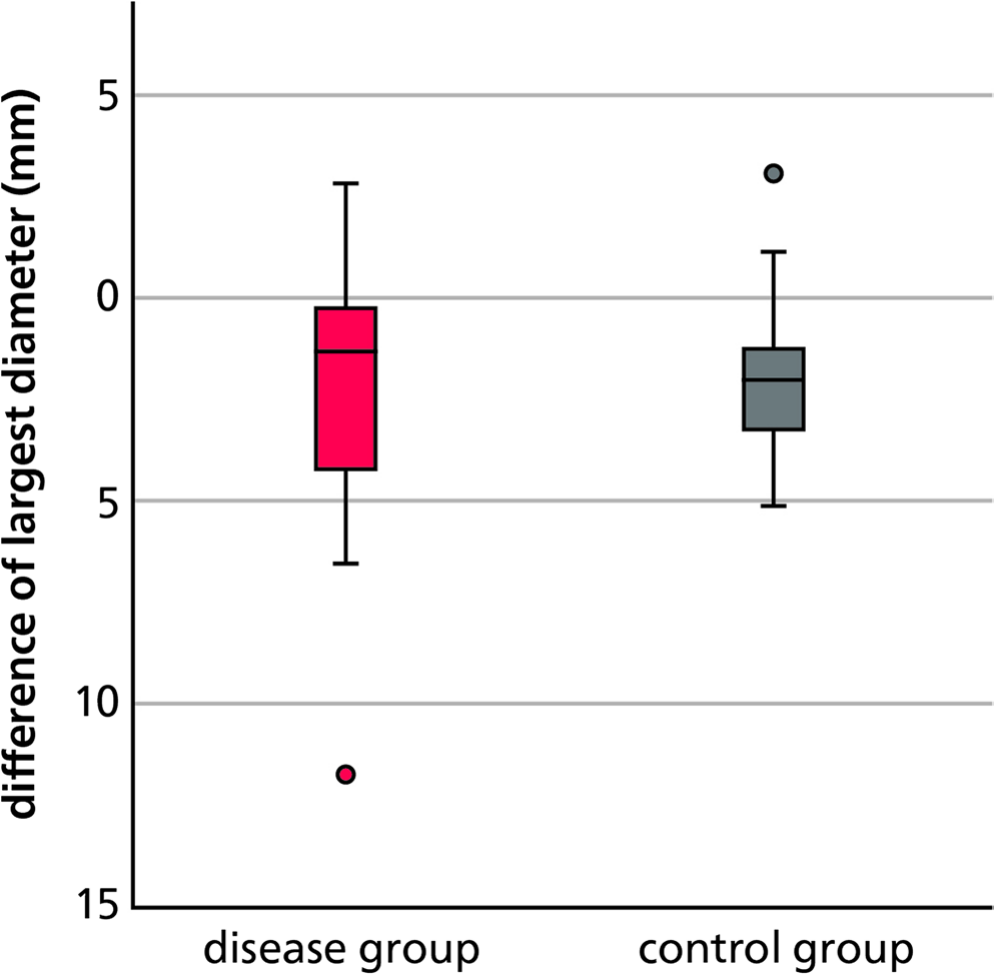
Change score in the osteoporosis (disease) and control groups

Subsequently, non-parametric analyses using Mann–Whitney U tests were performed to assess group differences. The test for the change score based on absolute lesion size revealed no statistically significant difference between the groups (*p* = 0.920). These findings are visualized in Fig. 3, which presents the distribution of absolute change scores in a boxplot, confirming the absence of significant intergroup variation.

Similarly, the Mann–Whitney U test conducted for the change score in PAI grades also showed no significant difference between the two groups (*p* = 1.000).

## Discussion

Osteoporosis is a systemic condition predominantly affecting the elderly and is characterized by reduced bone mineral density and compromised bone microarchitecture. While the relationship between osteoporosis and generalized skeletal bone loss is well established, its potential impact on the healing of periapical periodontitis following endodontic treatment remains insufficiently understood. The present study aimed to evaluate whether, and to what extent, osteoporosis influences periapical bone regeneration after root canal therapy.

The findings revealed no statistically significant differences in the radiographic healing outcomes of periapical lesions between osteoporotic patients and healthy controls. However, lesion size in the osteoporosis group showed, on average, approximately 20% less reduction compared to the control group. Both groups exhibited an average improvement of one grade on the Periapical Index (PAI). However, due to the exploratory nature and limited statistical power of the study, these findings should be interpreted with caution and cannot be generalized beyond this selective cohort.

### Connection between osteoporosis and oral bone density

In a study involving 41 healthy women aged 20 to 78 years, bone mineral density (BMD) was assessed in various anatomical regions using dual-energy X-ray absorptiometry (DXA) [13]. The results revealed significant correlations between the BMD of the maxilla and that of the mandible, lumbar spine, hip, and radius [13].

Payne et al. conducted a two-year longitudinal study involving 38 postmenopausal women, in which changes in alveolar bone height and density were analyzed in osteoporotic or osteopenic patients compared to women with normal lumbar spine BMD [14]. Computer-assisted evaluations demonstrated significantly greater losses in alveolar bone height and density in the osteoporotic/osteopenic group than in the control group with normal BMD. These findings support the hypothesis of a systemic association between skeletal and oral bone loss in osteoporotic individuals [14].

In another study involving 1,341 postmenopausal women aged 53 to 85 years, alveolar crest height and bone density were assessed using intraoral radiographs and DXA. Participants were stratified into osteoporosis severity categories based on their T-scores. Statistical analysis revealed a clear correlation between higher T-scores—indicating more advanced osteoporosis—and reduced alveolar crest height [7].

### Link between osteoporosis and tooth loss

The association between osteoporosis and tooth loss has been repeatedly described in the scientific literature [10]. Several studies indicate that individuals with osteoporosis may be at an increased risk of tooth loss compared to non-osteoporotic individuals [11, 12]. In a cross-sectional analysis of 1,914 participants aged 48 to 95 years, the relationship between the number of remaining teeth and bone mineral density (BMD) of the lumbar spine and femoral neck was assessed using dual-energy X-ray absorptiometry (DXA) [15]. A significant correlation was found between the number of remaining teeth and femoral neck BMD in both men and women, whereas no such association was observed with lumbar spine BMD [15]. Another study involving 651 women, including 140 diagnosed with osteoporosis, reported that osteoporotic individuals had, on average, at least one tooth fewer than non-osteoporotic women [16], suggesting a potential influence of systemic bone loss on oral health. In a further investigation of 333 patients, the association between the number of remaining teeth and osteoporotic status was examined while adjusting for potential confounders such as age, smoking, alcohol consumption, and hormone replacement therapy [3]. Although a significant correlation between osteoporosis and tooth loss was observed, the analysis indicated that other systemic factors may have a greater impact on dental health than osteoporosis alone [3]. Despite multiple studies suggesting an association between reduced bone mineral density and increased tooth loss in osteoporotic individuals, the overall body of evidence remains inconsistent. Promising insights also emerge from studies investigating the link between osteoporosis and periodontal disease. In a study of 90 women aged 45 to 70 years with chronic periodontitis, periodontal disease severity was assessed using clinical attachment level (CAL). DXA measurements demonstrated significantly greater CAL in osteoporotic participants compared to those with normal bone density [9]. These findings were corroborated by Brennan et al., who studied 1,329 postmenopausal women and found strong evidence of an association between osteoporosis and clinical attachment loss [8]. In contrast, another study found no clear association between edentulism, periodontal disease, and bone mineral density [17].

Osteoporosis and periodontitis are both chronic conditions characterized by progressive bone resorption and share a range of common risk factors, including advanced age, genetic predisposition, hormonal changes, smoking, and deficiencies in calcium and vitamin D intake. It has been hypothesized that these diseases not only exhibit similar pathophysiological mechanisms—such as altered bone metabolism and inflammatory responses—but may also act as mutual risk factors and influence one another reciprocally. This potential bidirectional relationship underscores the importance of an interdisciplinary treatment approach that addresses both conditions simultaneously [18]. Current evidence suggests a link between the metabolic changes associated with osteoporosis and their effects on tooth retention and periodontal health, although this association appears to be modest in some aspects. The majority of studies support an association between osteoporosis and an increased prevalence or severity of periodontitis [19]. Furthermore, a large cohort study demonstrated a significantly higher prevalence of periapical lesions in osteoporotic patients, with bisphosphonate therapy potentially associated with a reduced lesion frequency [11]. However, these findings could not be confirmed in the present study. A recent systematic review including three observational studies reported, despite methodological limitations, an increased prevalence of periapical lesions in osteoporotic patients in two studies, while one study found no difference between osteoporotic and non-osteoporotic individuals. The latter finding is consistent with the results of the present investigation [10].

### Limitations and strengths

It should be noted that the patient population in this study is relatively small, with a total of 26 subjects (13 in the osteoporosis group and 13 in the control group). Further studies with larger samples are therefore necessary to obtain reliable and generalizable results. Given the limited sample size, the statistical power of the study is restricted, and the report *p*-values must therefore be interpreted with caution, as the risk of a Type II error (false negative) is substantial. Accordingly, no formal a priori power analysis was performed, and the statistical analyses were intended to be exploratory and hypothesis-generating rather than confirmatory. The strict inclusion criteria and the requirement for complete follow-up imaging and documentation may have introduced selection bias, as patients included in the analysis might systemacially differ from those excluded, for example, with regard to thealth status or treatment compliance. The lack of classification according to the severity of osteoporosis, medication use, and time since diagnosis, including the distinction between primary and secondary osteoporosis, may have led to distortions and should be taken into account when interpreting the results. In addition, although patients with systemic diseases were excluded from the study to minimize bias, patients with non-systemic diseases were included in the analysis so as not to further reduce the sample size. This raises the question of whether comorbidities such as allergic rhinitis or depression had an influence on the results. Studies from 2018 suggest that depression increases the risk of periodontitis by 19% [20]. In addition to possible comorbidities, concomitant medications may also have influenced the study results, which should be taken into account in future studies, including sex, smoking status, minor systemic conditions, and concomitant medications. Variations by the practitioner and incomplete documentation of endodontic treatment protocols may have led to additional distortions and should be considered when interpreting the results. Another critical aspect is the accuracy of the radiological evaluation, as various sources of error can lead to potential misinterpretations. Artifacts, addition and subtraction effects, distortions, shifts, and blurring are known limitations of conventional radiological imaging. In addition, two-dimensional X-rays represent three-dimensional anatomical structures, which can lead to diagnostic limitations [12].

Periapical lesions may not always be visible on X-rays despite their clinical presence [21–23]. Another study showed that periapical inflammation could be detected histologically even though it appeared radiologically unremarkable [24]. These findings underscore the limitations of conventional X-ray diagnostics and the need for further imaging procedures for a more precise assessment of periapical changes. This limitation is particularly relevant in older patients with reduced bone density, as two-dimensional X-rays can potentially underestimate the extent of periapical lesions and their healing. The exclusive use of two-dimensional radiological imaging represents a further limitation, particularly with regard to the assessment of the quality of root fillings. Essential criteria such as length and homogeneity can only be estimated using conventional X-rays, but cannot be assessed with absolute certainty [25]. Nevertheless, radiological assessment currently remains the only established method for evaluating root filling quality. In addition, measurement inaccuracies and transmission errors cannot be completely ruled out when evaluating X-ray images. Given the inherent subjectivity of radiographic interpretation, all images were independently evaluated by two calibrated examiners. To minimize interobserver variability, a calibration process was conducted by an experienced assessor prior to the evaluation. This procedure aimed to ensure a high level of consistency and diagnostic reliability in radiographic assessment.

## Conclusions

Within the limitations of this exploratory retrospective controlled pilot cohort study, no statistically significant differences in radiographic periapical healing were observed between patients with osteoporosis and matched healthy controls. However, due to the limited sample size and restricted statistical significance, no definitive conclusions regarding the influence of osteoporosis on endodontic healing can be drawn. The present findings should therefore be interpreted with caution and primarily serve to inform the design of future studies.

## Data Availability

Data is contained within the article.
